# CMR quantification of aortic regurgitation in asymptomatic patients with significant aortic regurgitation: prediction of clinical outcome

**DOI:** 10.1186/1532-429X-13-S1-O99

**Published:** 2011-02-02

**Authors:** Saul G Myerson, Jo D'Arcy, John P Greenwood, Raad Mohiaddin, Theodoros D Karamitsos, Jane M Francis, Adrian P Banning, Jonathan P Christiansen, Stefan Neubauer

**Affiliations:** 1 University of Oxford Centre for Clinical Magnetic Resonance Research (OCMR), Oxford, UK; 2 University of Leeds, Leeds, UK; 3 CMR Unit, Royal Brompton Hospital and National Heart and Lung Institute, London, UK; 4 John Radcliffe Hospital, Oxford, UK; 5 North Shore Hospital, Auckland, New Zealand

## Introduction

The timing of valve surgery in asymptomatic patients with significant aortic regurgitation can be challenging. Current indications focus on symptoms and left ventricular (LV) dilation/dysfunction, but prognosis is already reduced by this time. Quantification of the regurgitation has not previously been used to guide management, likely due to the difficulty of achieving this with echocardiography. Cardiovascular magnetic resonance (CMR) can accurately quantify aortic regurgitation and LV volumes, and we examined whether either could predict symptom development and the need for aortic valve surgery.

## Methods

94 asymptomatic patients with moderate or severe aortic regurgitation on echocardiography were identified from four high volume CMR centres. CMR was performed to quantify aortic regurgitation and LV volume indices, and subsequent clinical follow up occurred for up to 7 years (mean 2.6 ±2.1 years). The best predictors of progression to symptoms and other conventional indications for surgery were determined.

## Results

**Figure 1:**
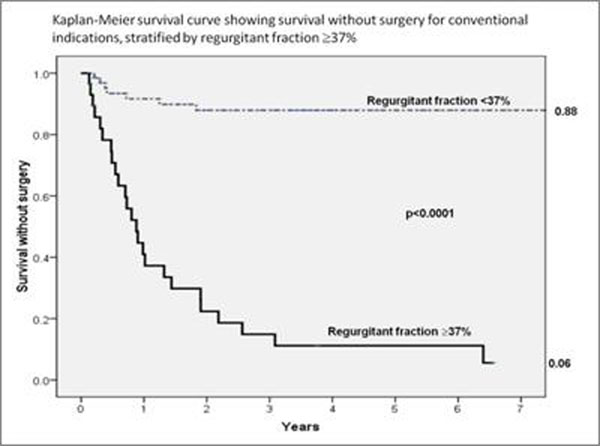


Aortic regurgitant fraction was the best predictor of clinical outcome; area under the curve (AUC) on receiver operating characteristics analysis 0.93 (p<0.0001), with a specificity of 93% and sensitivity of 78% for predicting the progression to symptoms and surgery. Survival without surgery was 88% for patients with a regurgitant fraction <37%, compared to 6% for those with a regurgitant fraction ≥37% (see figure 1). Regurgitant volume >38mls and regurgitant volume index >25mls/m^2^ were also good predictors (AUC 0.91 and 0.90 respectively), though regurgitant fraction had significantly greater predictive power (odds ratio 1.26 compared to 1.09 for regurgitant volume). LV volumetric indices also predicted outcome, but less strongly than measures of regurgitation: LV end-diastolic volume >267mls (AUC 0.85), LV end-systolic volume >88mls (AUC 0.78). Regurgitant fraction and volume were the only independent outcome predictors on multiple logistic regression analysis. The predictive ability of CMR applied to patients with both moderate and severe aortic regurgitation on echocardiography. Supporting data also comes from a comparison with patients already planning to undergo surgery at the time of CMR scanning, which showed similar regurgitant fractions in the surgical group (mean ±SD: 45.4 ±12.1%) compared to the initially asymptomatic patients who developed symptoms or other indications for surgery (42.8 ±10.4%); p=0.32. Subjects who remained asymptomatic had a significantly lower regurgitant fraction: 25.3 ±8.6% (p<0.0001 vs. both the planned surgical group and the symptom progression group)

## Conclusions

CMR quantification of aortic regurgitation and LV volumes accurately predicts the progression to symptoms/surgery. Its use in patients with aortic regurgitation should be encouraged.

